# Use of traditional medicine in middle-income countries: a WHO-SAGE study

**DOI:** 10.1093/heapol/czw022

**Published:** 2016-03-30

**Authors:** Oyinlola Oyebode, Ngianga-Bakwin Kandala, Peter J Chilton, Richard J Lilford

**Affiliations:** ^1^University of Warwick Medical School, Gibbet Hill Campus, Coventry CV4 7AL, UK; ^2^Department of Mathematics and Information sciences, Faculty of Engineering and Environment, Northumbria University, Newcastle upon Tyne, NE1 8ST, UK and; ^3^Health Economics and Evidence Synthesis Research Unit, Department of Population health Luxembourg Institute of Health, Strassen, Luxembourg

**Keywords:** Medicine, traditional, developing countries, health policy, World Health Organization

## Abstract

It is frequently stated in the scientific literature, official reports and the press that 80% of Asian and African populations use traditional medicine (TM) to meet their healthcare needs; however, this statistic was first reported in 1983. This study aimed to update knowledge of the prevalence of TM use and the characteristics of those who access it, to inform health policy-makers as countries seek to fulfil the WHO TM strategy 2014–23 and harness TM for population health. Prevalence of reported use of TM was studied in 35 334 participants of the WHO-SAGE, surveyed 2007–10. TM users were compared with users of modern healthcare in univariate and multivariate analyses. Characteristics examined included age, sex, geography (urban/rural), income quintile, education, self-reported health and presence of specific chronic conditions. This study found TM use was highest in India, 11.7% of people reported that their most frequent source of care during the previous 3 years was TM; 19.0% reported TM use in the previous 12 months. In contrast <3% reported TM as their most frequent source of care in China, Ghana, Mexico, Russia and South Africa; and <2% reported using TM in the previous year in Ghana, Mexico, Russia and South Africa. In univariate analyses, poorer, less educated and rural participants were more likely to be TM-users. In the China multivariate analysis, rurality, poor self-reported health and presence of arthritis were associated with TM use; whereas diagnosed diabetes, hypertension and cataracts were less prevalent in TM users. In Ghana and India, lower income, depression and hypertension were associated with TM use. In conclusion, TM use is less frequent than commonly reported. It may be unnecessary, and perhaps futile, to seek to employ TM for population health needs when populations are increasingly using modern medicine.

Key Messages

- Traditional medicine (TM) use is infrequent in China, Ghana, Mexico, Russia and South Africa and much less frequent than commonly reported in India.
- It may be unnecessary, and perhaps futile, to seek to harness TM for population health needs (as stipulated by the WHO Traditional Medicine Strategy 2014–23) when populations are demonstrating a preference for modern medicine.

## Introduction

It is frequently stated in the scientific literature (Stekelenburg *et al.* 2005; Tilburt and Kaptchuk 2008; Birhan *et al.* 2011; Mbatha *et al.* 2012; Sato 2012b; Gude 2013; Merriam 2013; Ekor 2014), official fact sheets and reports (WHO 2002, 2008; Kasilo *et al.* 2010) and the press (BBC News 2014; Modern Ghana 2014) that 80% of people in Asian and African countries (or sometimes that 80% of the world’s population) use traditional medicine (TM) practitioners to meet their primary healthcare needs. This statistic has also been used in policy-making and in defence of traditional, complementary and alternative medicine (King and Homsy 1997; UN 2009; ABC News 2014; Disabled-World 2014). However, when a piece of information becomes widely quoted it may become accepted without question and continue to be used, even though it has long been out of date. Kate Wilkinson traced the use of this statistic and found that it is likely to have originated in a *World Health Organisation* (WHO) textbook published in 1983, with the original data on which it was based now lost (Traditional Medicine and Health Care Coverage 1983; Africa Fact Check Blog 2014). More recent data suggest that the use of TM in some Asian and African countries is substantially lower and is on the decline (Peltzer 2009; Nxumalo 2011; Angmo 2012; Sato 2012a; Awiti 2014; Mee *et al.* 2014).

In low- and middle-income countries where the number of practitioners of modern medicine may not be enough to meet the health care needs of the country, TM and its practitioners are considered an important resource for population health. Compared to modern medicine, TM is perceived to be more affordable, accessible and acceptable to the communities in which it operates (Sato 2012b). Integration of TM and modern medicine has been recommended by the WHO since 1978 (WHO 2002). The recently published WHO Traditional Medicine Strategy 2014–23 has two key goals, one of which is to support Member States in harnessing the potential contribution of traditional and complementary medicine to health, wellness and people-centred health care (WHO 2013).

The extent to which the WHO goal can be realized will depend on the demand for TM services. Up-to-date knowledge of the prevalence of TM use and the characteristics of those who access this kind of health care is therefore necessary. We have examined these questions in survey data from six populous middle-income countries.

## Methods

### Participants and data

Study participants were adults aged 18 years and over who were part of the WHO Study on Global AGEing and Adult Health (SAGE) (available at http://www.who.int/healthinfo/sage/cohorts/en/index2.html). Participants were surveyed between 2007 and 2010 (Wave 1) in six middle-income countries: China, Ghana, India, Mexico, Russia and South Africa. SAGE used a clustered household sampling strategy designed to generate nationally representative cohorts of older people (over 50 years of age) with data collected on younger people for comparison. One household questionnaire was completed for each selected household in face-to-face interviews, and individual questionnaires were collected from one randomly selected individual aged 18–49 years and all individuals aged over 50 years (including by proxy where an individual was unable to complete the questionnaire). Individual response rates varied—53% in Mexico, 68% in India, 75% in South Africa, 81% in Ghana, 83% in Russia and 93% in China. Further details of SAGE have been published elsewhere (Kowal *et al.* 2012) Although the main interest of this article lies in examining use of TM in Asian and African countries, analysis of Mexican and Russian data was done for completeness, and for comparison. Participants were excluded from the study if they did not respond to questions on their health care use over the previous three years.

The SAGE study received human subjects testing and ethics council approval from the research review boards local to each participating site and from the WHO Ethical Review Committee. Written informed consent was obtained from each respondent before interview and examination. A standard consent form, approved by the WHO ethics review committee, was read to the respondent in the respondent’s language.

## Outcome variables

WHO-SAGE participants were asked two questions, which were used to examine use of TM. First, they were asked ‘Thinking about health care you needed in the last 3 years, where did you go “*most often*” when you felt sick or needed to consult someone about your health?’. Second, participants were asked questions relating to contact with health care providers over the last 12 months. If the participant reported that they had made contact with a health care provider in the last year, then they were asked ‘which was the health care provider you visited?’ and provided with a list of possible responses including the local terms for traditional healers. Each participant was asked about a maximum of three encounters with health professionals that occurred within the last 12 months. The results from these questions are recorded in Table 1.

**Table 1.** Characteristics of study participants [*n* (%) adjusted %] (unless otherwise indicated)

	China	Ghana	India	Mexico	Russia	South Africa
No. of participants	11 284	4661	9970	2346	3662	3411
**Age group**						
<40	485 (4.3) 29.8	380 (8.2) 39.7	2859 (28.7) 49.7	196 (8.4) 51.8	189 (5.2) 37.2	187 (5.5) 44.7
40–49	669 (5.9) 44.3	325 (7.0) 34.7	1264 (12.7) 25.1	167 (7.1) 21.7	138 (3.8) 21.5	125 (3.7) 31.2
50–59	4222 (37.4) 11.7	1531 (32.9) 10.4	2584 (25.9) 12.3	382 (16.3) 12.7	1188 (32.4) 18.7	1335 (39.2) 12.0
60–69	3100 (27.5) 8.2	1107 (23.8) 7.0	2007 (20.1) 7.7	819 (34.9) 6.8	927 (25.3) 10.2	992 (29.1) 7.4
70+	2808 (24.9) 6.0	1318 (28.3) 8.3	1256 (12.6) 5.2	775 (33.0) 7.0	1220 (33.3) 12.5	770 (22.6) 4.7
**Female**	6207 (55.0) 49.1	2260 (48.5) 50.4	6193 (62.1) 50.3	1477 (63.0) 52.0	2438 (66.6) 55.0	1988 (58.3) 52.8
**Income quintile**						
1 (poorest)	2087 (18.6) 9.9	883 (19.0) 15.1	1750 (17.6) 20.3	480 (20.5) 16.6	608 (16.6) 12.7	593 (17.5) 18.9
2	2135 (19.0) 15.9	907 (19.5) 18.2	1911 (19.3) 21.2	484 (20.7) 23.3	710 (19.4) 12.8	637 (18.8) 19.5
3	2234 (19.9) 18.3	920 (19.8) 19.0	1912 (19.3) 19.9	418 (17.8) 20.1	749 (20.5) 16.5	658 (19.4) 20.5
4	2402 (21.4) 23.4	979 (21.0) 22.4	2093 (21.1) 18.0	491 (21.0) 15.4	751 (20.5) 23.5	739 (21.8) 19.4
5 (richest)	2370 (21.1) 32.6	965 (20.7) 25.3	2252 (22.7) 20.6	470 (20.1) 24.6	840 (23.0) 34.5	763 (22.5) 21.8
**Education**						
Primary or less	6690 (59.3) 37.7	3424 (73.9) 63.4	7142 (71.6) 61.4	1804 (76.9) 51.7	370 (10.1) 3.2	2030 (70.6) 37.5
Secondary	2405 (21.3) 32.1	255 (5.5) 10.8	1196 (12) 15.7	245 (10.4) 23.5	663 (18.1) 10.9	429 (14.9) 27.1
Tertiary or more	2189 (19.4) 30.3	953 (20.6) 25.7	1632 (16.4) 23.0	297 (12.7) 24.8	2627 (71.8) 8.6	417 (14.5) 35.4
**Urban**	5526 (49.0) 48.7	1928 (41.4) 46.0	2583 (25.9) 25.7	1701 (72.5) 77.8	2776 (75.8) 81.5	2346 (68.9) 69.3
**Self-reported health**						
Very good	397 (3.5) 10.7	303 (6.5) 16.3	437 (4.4) 8.1	79 (3.4) 7.0	23 (0.6) 1.9	181 (5.3) 17.9
Good	3227 (28.6) 42.8	1748 (37.5) 47.3	3326 (33.4) 43.3	859 (36.6) 50.3	478 (13.1) 37.3	1107 (32.5) 44.5
Moderate	5165 (45.8) 35.2	1861 (39.9) 27.0	4547 (45.6) 37.3	1124 (47.9) 35.5	2120 (57.9) 49.4	1537 (45.1) 27.5
Bad	2225 (19.7) 10.4	646 (13.9) 7.4	1518 (15.2) 10.5	269 (11.5) 7.0	960 (26.2) 10.9	521 (15.3) 8.5
Very bad	257 (2.3) 1.0	102 (2.2) 1.8	141 (1.4) 0.8	15 (0.6) 0.2	77 (2.1) 0.5	59 (1.7) 1.5
**Arthritis**	4381 (38.9) 23.3	2042 (43.8) 25.8	3,795 (38.1) 26.5	788 (33.6) 24.0	886 (24.2) 32.0	1346 (39.5) 20.6
**Stroke**	616 (5.5) 1.6	194 (4.2) 2.1	403 (4.0) 3.0	237 (10.1) 7.9	349 (9.6) 5.0	228 (6.7) 2.8
**Angina**	1791 (15.9) 7.8	931 (20) 12.9	2979 (29.9) 24.2	335 (14.3) 10.4	1743 (47.8) 24.8	526 (15.4) 7.9
**Diabetes**	799 (7.1) 2.9	175 (3.8) 2.1	533 (5.4) 3.2	454 (19.4) 9.8	344 (9.4) 3.5	354 (10.4) 3.2
**COPD**	1669 (14.8) 6.8	211 (4.5) 3.0	1736 (17.4) 12.9	488 (20.8) 17.5	1169 (32.0) 20.4	307 (9.0) 5.4
**Asthma**	1771 (15.8) 8.3	333 (7.2) 4.7	1871 (18.8) 14.3	425 (18.1) 13.0	1019 (27.9) 14.8	370 (10.9) 5.4
**Depression**	1920 (17.1) 13.5	823 (17.7) 13.2	3423 (34.3) 28.7	856 (36.5) 32.9	1646 (45.1) 30.1	440 (12.9) 9.8
**Hypertension**	5069 (45.3) 29.7	2429 (52.1) 41.0	3006 (30.2) 23.7	1025 (43.7) 22.5	2470 (67.6) 40.9	2442 (71.7) 48.9
**Cataracts**	3830 (34.2) 14.2	1144 (24.6) 13.9	4829 (48.6) 36.2	1057 (45.2) 29.8	1446 (40.2) 21.0	288 (8.5) 3.4
**TM most frequent source of care**	24 (0.2) *0.3*	123 (2.6) *2.1*	984 (9.9) *11.7*	6 (0.3) *0.2*	6 (0.2) *0.03*	40 (1.2) *1.7*
**1+ health consultation in previous 12m**	6716 (59.5) 45.9	2971 (63.7) 52.0	8458 (84.8) 68.7	1001 (42.7) 31.5	2594 (70.8) 58.3	2072 (60.7) 38.1
**1+ TM consultation in previous 12m**	666 (5.9) *9.4*	123 (2.6) *1.5*	1852 (18.6) *19.0*	3 (0.1) *0.1*	6 (0.2) *0.04*	3 (0.1) *0.02*
**Total reported consultations**	13 843	5868	17 939	2349	5243	4912
**TM Consultations** **[*n*, (% of total reported consultations)]**	1175 (8.5)	181 (3.1)	3589 (20.0)	4 (0.2)	6 (0.1)	3 (0.1)
**Consultation cost to household** **[u, (SD)]**	214.63 (1199.99)	156 959.9 (774 203.9)	441.49 (1722.07)	671.56 (4188.36)	9303.35 (233 483.9)	119.91 (216.89)

To further examine the characteristics of TM users, we chose to use the latter question only, this is because it is likely to be less vulnerable to recall bias, as it examines the last 12 months, rather than the last 3 years. We therefore classified anyone who reported at least one consultation with a TM practitioner in the last 12 months as a TM user. For comparison we defined those who had at least one contact with a health care provider in the last 12 months, but who did not report contact with a TM practitioner as modern health care users.

## Other variables

Participant characteristics examined included sex and geography (urban or rural) analysed as binary variables; income quintile, education (grouped as: primary or less; secondary; tertiary or more) and self-reported health (very good; good; moderate; bad; very bad) analysed as ordinal categorical variables; and age (grouped as: <40; 40–49; 50–59; 60–69; 70+) analysed in the univariate analysis both as an ordinal categorical variable and as a nominal variable (see Statistical analysis section below), and in the multivariate analysis as a categorical variable with age 70+ as the reference category.

Presence of one of a list of chronic diseases identified by the survey was also examined as a participant characteristic. These were: arthritis, stroke, angina, diabetes, chronic obstructive pulmonary disease, asthma, depression, hypertension and cataracts. For each chronic disease examined, except diabetes, there were questions relating to participant-reported doctor diagnosis, alongside data items allowing recording of probable undiagnosed disease. Two examples are given in Box 1 below.

Box 1.

Asthma

1. Have you ever been diagnosed with asthma (an allergic respiratory disease)?
2. During the last 12 months have you experienced any of the following:
  - Attacks of wheezing or whistling breathing?
  - Attack of wheezing that came on after you stopped exercising or some other physical activity?
  - A feeling of tightness in your chest?
  - Have you woken up with a feeling of tightness in your chest in the morning or any other time?
  - Have you had an attack of shortness of breath that came on without obvious cause when you were not exercising or doing some physical activity?

Stroke

1. Have you ever been told by a health professional that you have had a ‘stroke’?
2. Have you ever suffered from ‘sudden onset’ of paralysis or weakness in your arms or legs on ‘one side’ of your body for >24 h?
3. Have you ever had for >24 h ‘sudden onset’ of loss of feeling on ‘one side’ of your body without anything having happened to you immediately before?

In order to identify undiagnosed hypertension, three blood pressure readings were taken from all participants. Hypertension was defined as an average systolic blood pressure over 140 or average diastolic blood pressure over 90. There were no questions or objective measurements taken in order to identify undiagnosed diabetes, therefore only diagnosed diabetes has been examined here.

Finally, we examined costs of consultation with a health care provider. Participants who reported contact with a health care provider in the last 12 months were asked how much they, or their household, paid in relation to this contact. Costs were analysed as continuous variables in the local currency in which they were recorded.

## Statistical analysis

Descriptive statistics are used to characterize the study population. Survey weights were used for these to give results representative of the general national populations from which the study populations were drawn.

To examine the association between our variables of interest and use of TM we did univariate and multivariate analyses. Univariate analyses were carried out using the Pearson correlation co-efficient if the independent variable was ordinal categorical; Fisher’s exact test if the independent variable was binary; and Pearson’s χ^2^ test alongside Pearson’s correlation co-efficient for age-group to address whether age was associated with use of TM in a non-linear fashion.

Multivariate logistic regression was used to examine the independent association of the variables of interest with use of TM. All those variables that were significantly associated with TM use in univariate analyses were included in the models. Due to significant correlation between education, income quintile and geography, education was dropped from the model to reduce collinearity. Survey weights were not used in these analyses. Data were analysed in STATA/SE version 13.

## Results

The study included 35 334 participants after 4857 (12.1%) were excluded due to missing data on their health care use over the previous three years. Of these, 23 851 (67.5%) participants reported at least one contact with health services in the previous 12 months. A total of 50 154 consultations were discussed in interviews. Table 1 presents the characteristics of the participants in each country.

When asked where they went most frequently over the previous 3 years when they felt sick or needed to consult someone about their health, <1% of participants in China, Mexico and Russia reported going to a TM practitioner. Just 40 (1.7%) participants in South Africa and 123 (1.5%) participants in Ghana reported that they had used TM (percentages adjusted for survey design, therefore nationally representative). In contrast, 984 (11.7%) of participants in India reported that they most frequently visited traditional healers when they felt sick or needed to consult someone about their health (Table 1).

The number of participants who reported at least one TM consultation over the previous 12 months was higher than the number reporting that TM was their most frequent source of care over the past 3 years in China (666 participants, 9.4%) and in India (1852 participants, 19.0%), but lower in South Africa (3 participants, 0.02%). The percentage of all consultations reported by participants that were with a practitioner of TM varied from <1% in Mexico, Russia and South Africa, 3.1% in Ghana, 8.5% in China, to 20.0% in India (Table 1).

Univariate analyses, examining characteristics of people who reported using TM over the previous 12 months compared with those who reported other medical contact in the previous 12 months, were only conducted in datasets from China, Ghana and India, as the number of people who reported using TM over the previous 12 months in Mexico, Russia and South Africa were too low to make any meaningful conclusions. These results are presented in Table 2.

**Table 2.** Characteristics of users of traditional medicine by study characteristics

	China			Ghana			India		
	Modern medicine	Traditional medicine	*P*	Modern medicine	Traditional medicine	*P*	Modern medicine	Traditional medicine	*P*
**Age group**			0.009			0.041			0.684
<40	253 (4.2)	27 (4.0)	χ^2^ <0.001	226 (8.0)	4 (3.3)	χ^2^ 0.365	1890 (28.6)	512 (27.7)	χ^2^ 0.222
40–49	314 (5.2)	77 (11.5)		196 (6.9)	6 (4.9)		819 (12.4)	259 (14.0)	
50–59	2282 (37.7)	232 (34.7)		864 (30.4)	39 (31.7)		1681 (25.5)	491 (26.5)	
60–69	1662 (27.5)	176 (26.3)		677 (23.8)	29 (23.6)		1354 (20.5)	351 (19.0)	
70+	1544 (25.5)	157 (23.5)		879 (30.9)	45 (36.6)		862 (13.1)	239 (12.9)	
**Sex**			0.623			0.408			0.684
Male	2654 (43.8)	300 (44.8)		1366 (48.0)	64 (52.0)		2466 (37.3)	701 (37.9)	
Female	3401 (56.2)	369 (55.2)		1478 (52.0)	59 (48.0)		4140 (62.7)	1151 (62.2)	
**Income quintile**			<0.001			<0.001			<0.001
1 (poorest)	986 (16.4)	117 (17.8)		430 (15.2)	24 (19.5)		932 (14.1)	508 (27.4)	
2	1079 (17.9)	130 (19.7)		521 (18.4)	25 (20.3)		1122 (17)	502 (27.1)	
3	1166 (19.3)	142 (21.6)		578 (20.4)	43 (35)		1306 (19.8)	348 (18.8)	
4	1345 (22.3)	185 (28.1)		632 (22.3)	24 (19.5)		1528 (23.2)	250 (13.5)	
5 (richest)	1456 (24.1)	85 (12.9)		677 (23.9)	7 (5.7)		1701 (25.8)	243 (13.1)	
**Education**			<0.001			0.009			<0.001
Primary or less	3500 (57.8)	485 (72.5)		2032 (71.8)	101 (82.8)		4579 (69.3)	1488 (80.4)	
Secondary	1297 (21.4)	130 (19.4)		173 (6.1)	5 (4.1)		813 (12.3)	189 (10.2)	
Tertiary or more	1258 (20.8)	54 (8.1)		627 (22.1)	16 (13.1)		1214 (18.4)	175 (9.5)	
**Geography**			<0.001			0.016			<0.001
Urban	3048 (50.3)	87 (13)		1265 (44.5)	41 (33.3)		2047 (31)	176 (9.5)	
Rural	3007 (49.7)	582 (87)		1579 (55.5)	82 (66.7)		4559 (69)	1676 (90.5)	
**Self-reported health**			<0.001			0.015			0.115
Very good	212 (3.5)	11 (1.7)		198 (6.7)	9 (7.3)		273 (4.1)	89 (4.8)	
Good	1720 (28.4)	129 (19.3)		946 (33.3)	25 (20.3)		2146 (32.5)	622 (33.6)	
Moderate	2813 (46.5)	292 (43.7)		1180 (41.5)	58 (47.2)		3152 (47.7)	753 (40.7)	
Bad	1156 (19.1)	208 (31.1)		441 (15.5)	26 (21.1)		949 (14.4)	353 (19.1)	
Very bad	146 (2.4)	28 (4.2)		78 (2.7)	5 (4.1)		85 (1.3)	35 (1.9)	
**Arthritis**			<0.001			0.46			0.085
Yes	2506 (41.4)	336 (50.2)		1304 (45.9)	61 (49.6)		2636 (39.9)	698 (37.7)	
No	3546 (58.6)	333 (49.8)		1538 (54.1)	62 (50.4)		3970 (60.1)	1154 (62.3)	
**Stroke**			0.606			0.077			0.469
Yes	359 (5.9)	43 (6.4)		129 (4.5)	10 (8.1)		281 (4.3)	71 (3.8)	
No	5690 (94.1)	625 (93.6)		2712 (95.5)	113 (91.9)		6325 (95.8)	1781 (96.2)	
**Angina**			0.476			0.653			0.005
Yes	1010 (16.7)	104 (15.6)		600 (21.1)	28 (22.8)		2099 (31.8)	525 (28.4)	
No	5032 (83.3)	564 (84.4)		2241 (78.9)	95 (77.2)		4507 (68.2)	1327 (71.7)	
**Diabetes**			<0.001			0.522			<0.001
Yes	509 (8.4)	24 (3.6)		141 (5.0)	4 (3.25)		427 (6.5)	63 (3.4)	
No	5532 (91.6)	644 (96.4)		2700 (95.0)	119 (96.8)		6179 (93.5)	1789 (96.6)	
**COPD**			0.166			0.817			0.232
Yes	963 (15.9)	120 (18.0)		115 (4.1)	4 (3.25)		1213 (18.4)	317 (17.1)	
No	5087 (84.1)	547 (82.0)		2726 (96.0)	119 (96.8)		5393 (81.6)	1535 (82.9)	
**Asthma**			0.258			0.725			0.104
Yes	932 (15.5)	92 (13.8)		210 (7.4)	10 (8.13)		1312 (19.9)	336 (18.1)	
No	5095 (84.5)	577 (86.3)		2631 (92.6)	113 (91.9)		5294 (80.1)	1516 (81.9)	
**Depression**			0.169			<0.001			<0.001
Yes	1114 (18.4)	108 (16.2)		458 (16.1)	38 (30.9)		2251 (34.1)	768 (41.5)	
No	4933 (81.6)	560 (83.8)		2385 (83.9)	85 (69.1)		4355 (65.9)	1084 (58.5)	
**Hypertension**			0.001			0.007			<0.001
Yes	2910 (48.4)	276 (41.6)		1570 (55.2)	83 (67.5)		2139 (32.4)	465 (25.1)	
No	3101 (51.6)	388 (58.4)		1273 (44.8)	40 (32.5)		4467 (67.6)	1386 (74.9)	
**Cataracts**			<0.001			0.678			<0.001
Yes	2106 (35.1)	172 (25.9)		759 (26.7)	35 (28.5)		3235 (49.1)	995 (53.8)	
No	3902 (65.0)	493 (74.1)		2083 (73.3)	88 (71.5)		3348 (50.9)	855 (46.2)	
**Total cost to household**			0.144			0.843			<0.001
Mean (SD)	220.37 (1221.72)	147.37 (855.52)		157 792.8 (787 230.3)	143 173.9 (364 428.2)		518.79 (1943.90)	183.80 (371.92)	

COPD, chronic obstructive pulmonary disease. Where the independent variable is ordinal, *P* values calculated using Pearson’s correlation co-efficient. Where the independent variable is binary, using Fisher’s exact test. Where the independent variable is continuous, using t-test. Where the independent variable is nominal categorical, χ^2^. For the association between age group and use of TM both Pearson’s correlation co-efficient and χ^2^ are presented.

In the China, Ghana and India univariate analyses, income quintile, education and geography were associated with use of TM, with poorer, less educated and rural participants more likely to report use of TM in the last 12 months (Table 2).

In China, age group and self-reported health, as well as the presence of arthritis, diabetes, hypertension and cataracts were also associated with use of TM. Users of TM were younger, had worse self-reported health, and were also more likely to have arthritis; however, they were less likely to have hypertension, cataracts or doctor-diagnosed diabetes (Table 2).

In Ghana, univariate analyses showed that older participants were more likely to use TM. As in China, they had worse self-reported health. Hypertension showed the opposite association to the China data, i.e. those using TM were more likely to have high blood pressure. Users of TM were also more likely to have depression (Table 2).

In India, univariate analysis showed that although age group and self-reported health were not associated with use of TM, there were associations with several of the diseases examined. Depression and cataracts were more common in those treated with TM, whereas angina, hypertension and doctor-diagnosed diabetes were more prevalent in those using modern medicine. In addition, the total cost to the household was lower for users of TM in India (R183.80) than for users of modern medicine (R518.8) (there was no association seen with total cost of consultation and use of TM in China or Ghana) (Table 2).

Multivariate analysis examined those who had reported use of TM in the previous 12 months compared with those who had any other medical contact in the previous 12 months. There was a high degree of correlation between geography, income quintile and education in the three countries studied. For this reason, education was excluded from the multivariate analyses to reduce multi-collinearity (Table 3).

**Table 3.** Adjusted odd ratios and 95% confidence interval (CI) of users of traditional healers by the study characteristics

China		
	OR (CI)	*P*- value
Rural^a^	6.9 (5.4–8.9)	<0.001
Income quintile	1.2 (1.1–1.2)	<0.001
Self-reported health	1.5 (1.4–1.7)	<0.001
Arthritis	1.4 (1.2–1.7)	<0.001
Diabetes	0.6 (0.4–0.9)	0.028
Hypertension	0.8 (0.7–1)	0.039
Cataracts	0.6 (0.5–0.8)	<0.001
<40^b^	0.8 (0.5–1.3)	0.395
40–49^b^	1.7 (1.2–2.4)	0.002
50–59^b^	0.7 (0.5–0.9)	0.002
60–69^b^	0.8 (0.6–1)	0.093

Ghana		
	OR (CI)	*P*- value

Rural^a^	1.4 (1–2.2)	0.077
Income quintile	0.8 (0.7–0.9)	0.002
Self-reported health	1.1 (0.9–1.4)	0.27
Depression	2.2 (1.4–3.3)	<0.001
Hypertension	1.7 (1.2–2.5)	0.007
<40^b^	0.5 (0.2–1.4)	0.204
40–49^b^	0.8 (0.3–1.9)	0.623
50–59^b^	1.1 (0.7–1.7)	0.767
60–69^b^	0.9 (0.6–1.5)	0.74

India		
	OR (CI)	*P*- value

Rural^a^	1.3 (0.9–2)	0.217
Income quintile	0.8 (0.7–0.9)	0.001
Angina	1.0 (0.6–1.5)	0.838
Diabetes	0.8 (0.3–2.2)	0.644
Depression	2.4 (1.5–3.6)	<0.001
Hypertension	1.8 (1.2–2.7)	0.005
Cataracts	0.9 (0.6–1.4)	0.665
Total costs	1.0 (1.0–1.0)	0.949

^a^Geography was entered as a categorical variable with urban as the reference category.

^b^Age group was entered as a categorical variable with age 70+ as the reference category.

In the multivariate analysis of data from China, rurality was associated with use of TM. Worsening self-reported health and prevalence of arthritis were also associated with use of TM. TM users were less likely to have doctor-diagnosed diabetes and hypertension and cataracts were less prevalent in TM users. Age was associated with use of TM, with 40–49 year olds more likely to use TM and 50–59 year olds less likely to use TM compared with the over 70s. The association between income quintile and use of TM was reversed in this multivariate analysis, with increasing income associated with increasing use of TM (Table 3).

In Ghana and India, results were very similar. Rurality was not associated with use of TM. Increasing income was associated with reduced use of TM. Depression and hypertension were both more prevalent in users of TM. Age was not associated with use of TM in the Ghana analysis (and not included as a variable in the India analysis). Presence of angina, diabetes and cataracts and total cost of consultation were not associated with TM use in the India analysis (and not included as variables in the Ghana analysis) (Table 3).

## Discussion

This study has found that use of TM in six populous middle-income countries is much lower than has previously been reported. The country with the greatest reported use of TM is India, where 11.7% of people reported that their most frequent source of care was TM and 19.0% of people reported at least one consultation with a TM practitioner in the previous 12 months. In contrast, <3% reported using TM as their most frequent source of care in China, Ghana, Mexico, Russia and South Africa, and <2% reported using TM in the last 12 months in Ghana, Mexico, Russia and South Africa. Those who do make use of TM are more likely to be socio-economically disadvantaged.

Although use of TM is particularly low in the two sub-Saharan African countries examined, its use is more prevalent in China and India where the percentage use represents a very large population in absolute terms. Chinese TM is a point of pride for the Chinese Government. There is widespread belief that it works and it is part of the history, culture and politics of the country (Goss *et al.* 2014). Similarly in India, the government and the community may give certain traditional forms of medicine considerable respect, in terms of policies and funding. Further, in both China and India many physicians have training in traditional medicine and use traditional remedies as part of their treatment recommendations (Hesketh and Zhu 1997; van Gameren 2010; Kay 2013) Even so, the use of TM for healthcare in China and India is still considerably lower than commonly cited.

We are not the first to make the observation that use of TM is lower than the 80% commonly reported by the WHO and others, since a number of single country studies corroborate our findings. Analysis of nationally representative South African population-based surveys from 2005 to 2007 found <0.1% of the population had used TM in the past month (down from a high of 12.7% a decade earlier Peltzer 2009). A 2008 survey of households in South Africa (*n* = 4762) found that only 1.2% of respondents reported using traditional healers (Nxumalo *et al.* 2011). A household survey in Ghana (*n* = 4713) found that 83% used modern medicine as their first choice when they had need for health services, whereas only 12% chose traditional care, of which 5.5% pursued self-care through traditional methods and 6.5% consulted a traditional healer (Sato 2012a). In the Kenya Integrated Household and Budget Survey just 7.6% of respondents consulted ‘non-modern’ health care providers of which 0.2% visited a traditional healer (Awiti 2014). Angmo *et al.* (2012) reported that in Ladakh, India the number of traditional healers has fallen and the majority of the remaining practitioners are aged over 51. The study found that younger generations preferred other professions and there are areas where no apprentice healers were in training (although note that in our analysis no particular age-group had greater use of TM Angmo *et al.* 2012).

Similar to our findings in Ghana and India, others have found that those of a lower socio-economic status, who were unemployed, lived in rural areas and reported low health status were more likely to report use of traditional healers (van Gameren 2010; Nxumalo *et al.* 2011; Sato 2012a; Awiti 2014). Whether this is the most appropriate or simply the most accessible care for these marginalized groups needs further investigation.

Our results give some indication that traditional medicine is used as adjuvant therapy to modern medicine in China and India where more respondents stated that they had used traditional medicine in the last 12 months than answered that it was their main source of care.

In Ghana and India we found that depression was more prevalent among users of TM, and in China and Ghana self-reported health was lower among users of TM. A 2003 study in Tanzania found that the prevalence of mental disorders among patients of traditional healer centres was approximately twice that of patients attending primary health care clinics (Ngoma 2003). Interpretations of this may include the idea that when modern medicine leaves something to be desired, as with some mental illness, traditional medicine provides additional support. However, a national survey of 3651 South African adults between 2002 and 2004 found that of those with DSM-IV diagnoses for common mood, anxiety, and substance use disorders, just 9% had consulted traditional healers, 11% had consulted religious of spiritual advisors and 29% had consulted a modern medicine practitioner (Sorsdahl *et al.* 2009). In addition, a study of psychiatric patients in Gujarat, India were largely dissatisfied with their experience of TM, and those treated by both TM and modern medicine asserted that they would recommend modern medicine over TM (Schoonover *et al.* 2014).

The strengths of our study are that we examined data from six populous middle-income countries, including two Asian and two Sub-Saharan African countries. TM use was ascertained by whether participants reported a visit to a traditional healer within the last 12 months. This time limit allows current behaviour to be examined, rather than what participants may have done in earlier periods of their life, as well as reducing recall bias.

A limitation of our article is that due to the small numbers of people utilizing TM it was not possible to draw conclusions about the characteristics associated with use of TM in Mexico, Russia or South Africa. Further limitations include that it is based on reported, not observed behaviour, and therefore subject to reporting bias, for example: It may be that someone who had visited a doctor practising a government accredited system of traditional medicine such as Ayurveda in India would not have considered that they had visited an traditional practitioner or there may be reluctance to report TM use as Western education and lifestyles are seen as progressive i.e. social desirability bias could influence reporting. However, it is unlikely that this alone could account for the considerable distance of these figures from the 80% commonly reported. In addition, the questionnaire was not specifically designed to answer this question: it is a general survey covering a range of health-related topics and may not have probed this issue as carefully as a study designed to answer this particular question. However, taken in combination with observations made in single countries, it is hard to escape the conclusion that TM use is on the decline and the drop in use seems quite precipitate.

In conclusion, this study suggests that TM use in the countries studied is considerably lower than commonly reported. While our study documents the extent of TM use, it cannot provide an answer as to what motivates its continued use: whether traditional healers are used only when modern medicine is unavailable or unaffordable; or whether they continue to be used because they provide effective and acceptable treatments for some conditions. Other factors that may contribute to the decline in use of TM include changes in social trends and cultural beliefs and the political support and provision of resources for training, practising and increasing public awareness of modern medicine. Perhaps the policy position adopted by the WHO and others should be more nuanced, not encouraging TM use for health needs (e.g. malaria, angina) where use of TM is declining and where there are reasons to doubt its effectiveness in comparison to modern medicine. Instead further research should focus on understanding what role TM can play in improving population health and wellbeing.
